# Immune cell profile of sentinel lymph nodes in patients with malignant melanoma – FOXP3^+^ cell density in cases with positive sentinel node status is associated with unfavorable clinical outcome

**DOI:** 10.1186/1479-5876-11-43

**Published:** 2013-02-18

**Authors:** Anita Mohos, Tímea Sebestyén, Gabriella Liszkay, Vanda Plótár, Szabolcs Horváth, István Gaudi, Andrea Ladányi

**Affiliations:** 1 1st Institute of Pathology and Experimental Cancer Research, Semmelweis University, Budapest, Hungary; 2 Department of Pathology, St. John’s Hospital, Budapest, Hungary; 3 Department of Dermatology, National Institute of Oncology, Budapest, Hungary; 4 Department of Surgical and Molecular Pathology, National Institute of Oncology, 7-9. Ráth György u., Budapest, H-1122, Hungary; 5 National Cancer Registry, National Institute of Oncology, Budapest, Hungary

**Keywords:** Melanoma, Sentinel lymph node, Activated T cell, Regulatory T cell, Dendritic cell, Immunohistochemistry

## Abstract

**Background:**

Besides being a preferential site of early metastasis, the sentinel lymph node (SLN) is also a privileged site of T-cell priming, and may thus be an appropriate target for investigating cell types involved in antitumor immune reactions.

**Methods:**

In this retrospective study we determined the prevalence of OX40^+^ activated T lymphocytes, FOXP3^+^ (forkhead box P3) regulatory T cells, DC-LAMP^+^ (dendritic cell-lysosomal associated membrane protein) mature dendritic cells (DCs) and CD123^+^ plasmacytoid DCs by immunohistochemistry in 100 SLNs from 60 melanoma patients. Density values of each cell type in SLNs were compared to those in non-sentinel nodes obtained from block dissections (n = 37), and analyzed with regard to associations with clinicopathological parameters and disease outcome.

**Results:**

Sentinel nodes showed elevated amount of all cell types studied in comparison to non-sentinel nodes. Metastatic SLNs had higher density of OX40^+^ lymphocytes compared to tumor-negative nodes, while no significant difference was observed in the case of the other cell types studied. In patients with positive sentinel node status, high amount of FOXP3^+^ cells in SLNs was associated with shorter progression-free (P = 0.0011) and overall survival (P = 0.0014), while no significant correlation was found in the case of sentinel-negative patients. The density of OX40^+^, CD123^+^ or DC-LAMP^+^ cells did not show significant association with the outcome of the disease.

**Conclusions:**

Taken together, our results are compatible with the hypothesis of functional competence of sentinel lymph nodes based on the prevalence of the studied immune cells. The density of FOXP3^+^ lymphocytes showed association with progression and survival in patients with positive SLN status, while the other immune markers studied did not prove of prognostic importance. These results, together with our previous findings on the prognostic value of activated T cells and mature DCs infiltrating primary melanomas, suggest that immune activation-associated markers in the primary tumor may have a higher impact than those in SLNs on the prognosis of the patients. On the other hand, FOXP3^+^ cell density in SLNs, but not in the primary tumor, was found predictive of disease outcome in melanoma patients.

## Background

The sentinel lymph node (SLN), the first proximal lymph node draining a primary tumor, is a preferential site of early metastasis. Identification, removal and pathologic examination of SLNs are part of the routine surgical management in the case of melanoma and breast cancer. In melanoma, pathologic determination of the extent of SLN involvement provides a powerful prognostic indicator of survival [1].

Besides its importance in staging and prognosis, the sentinel node is also a privileged site of T-cell priming, and may thus be an appropriate target for investigating cell types and mechanisms involved in antitumor immune reactions. Previous studies have found evidence for alterations in immunoreactivity of SLNs compared either to non-sentinel lymph nodes (NSLNs) from the same patient cohort [2-7] or to control lymph nodes from non-tumor patients [7-10]. The results of these investigations, however, are somewhat controversial (Table 1). While some reports on melanoma and breast cancer suggested that sentinel lymph nodes are immune suppressed compared to non-sentinel nodes, containing lower number of DCs and T lymphocytes [2,3,7], other studies on various tumor types did not find such differences [4-6,11]. Similar controversy exists when SLNs are compared to non-tumor control lymph nodes [7-10,12].

**Table 1:** Prevalence of immune cell types in sentinel vs. non-sentinel nodes and in positive vs. negative SLNs

**Study (reference)**	**Tumor**	**Number of**	**Number of**	**Dendritic cells**		**Mature DCs**		**Plasmacytoid DCs**		**T cells**		**Regulatory T cells**	
	**type**	**patients (SLNs)**	**+ / - SLNs**	**SLN vs. NSLN**	**+ vs. - SLN**	**SLN vs. NSLN**	**+ vs. - SLN**	**SLN vs. NSLN**	**+ vs. - SLN**	**SLN vs. NSLN**	**+ vs. - SLN**	**SLN vs. NSLN**	**+ vs. - SLN**
*Compared to NSLNs of the same patient cohort*													
Cochran et al. 2001 [3]	melanoma	11 (21)	10 / 11	lower	NT	NT	NT	NT	NT	NT	NT	NT	NT
Botella-Estrada et al. 2005 [4]	melanoma	10 (17)	1 / 16	higher	NT	no diff.	NT	NT	NT	NT	NT	NT	NT
Gerlini et al. 2007 [20]	melanoma	27 (39)	8 / 31	NT	NT	NT	NT	NT	higher	NT	NT	NT	NT
Speeckaert et al. 2011 [14]	melanoma	116 (116)	26 / 90	NT	NT	NT	NT	NT	NT	NT	NT	NT	higher
Ma et al. 2012 [15]	melanoma	84 (84)	31 / 53	NT	lower	NT	higher	NT	NT	NT	NT	NT	higher
Huang et al. 2000 [2]^2^	breast cc.	21 (21)	not spec.	lower	NT	lower	NT	NT	NT	lower	NT	NT	NT
Kohrt et al. 2005 [7]^2^	breast cc.	29 (29)	29 / 0	lower	NT	NT	NT	NT	NT	lower (CD4^+^)	NT	NT	NT
Bembenek et al. 2008 [6]	breast cc.	79 (114)	51 / 28^3^	NT	NT	higher	NT	NT	NT	NT	NT	NT	NT
Ishigami et al. 2003 [11]	gastric cc.	27 (27)	8 / 19	no diff.	no diff.	NT	NT	NT	NT	no diff.	no diff.	NT	NT
Lee et al. 2011 [13]	gastric cc.	64 (64)	45 / 19^3^	NT	NT	NT	no diff.	NT	NT	NT	no diff.	NT	higher
Sakakura et al. 2005 [5]	oral cc.	12 (41)	0 / 41	higher	NT	no diff.	NT	NT	NT	NT	NT	NT	NT
*Compared to non-tumor control nodes*													
Mansfield et al. 2011 [10]	melanoma	20 (20)	8 / 12	NT	NT	lower	no diff.	no diff.	no diff.	lower (CD8^+^)	no diff.	no diff.	no diff.
Poindexter et al. 2004 [12]	breast cc.	50 (50)	25 / 25	no diff.	no diff.	no diff.	lower	NT	NT	NT	NT	NT	NT
Mansfield et al. 2009, 2011 [8,9]	breast cc.	47 (47)	36 / 11	no diff.	no diff.	higher	lower	no diff.	no diff.	higher (CD8^+^)	no diff.	no diff.	higher

Columns SLN vs. NSLN: comparison of negative SLNs to negative NSLNs or controls (except in studies [2] and [7]); columns + vs. - SLN: comparison of positive to negative SLNs.

^1^SLNs and NSLNs of unspecified status were compared; ^2^positive SLNs were compared to mixed (+ and -) NSLNs; also compared to non-tumor controls, with similar results

^3^number of patients with + / - SLN status; NT: not tested;no diff.: no difference; not spec.: not specified.

Comparison of immune parameters in tumor-positive vs. -negative SLNs also yielded somewhat ambiguous data (Table 1). Generally, sentinel nodes with metastases were demonstrated to contain more FOXP3^+^ cells than tumor-free ones in melanoma, breast or gastric cancer [8,13-15]. Other T-cell subsets were less frequently studied but were mostly found in similar amount in positive and negative SLNs. Data concerning dendritic cells infiltrating various cancers were less conclusive, with some studies showing lower amount of certain DC subtypes in tumor-containing than in tumor-free SLNs [9,12] while others reporting no such difference [10,11,13].

While the detection of metastases in SLN has an important role in determining the prognosis of patients in the case of several tumor types, the potential of prognostic application of the examination of immune status of these nodes is largely unexploited. A report by Cochran et al. demonstrated the prognostic role of DC density in melanoma SLNs [16], while data from studies by Elliott and coworkers indicated an association between survival of melanoma patients and the density of DC-LAMP^+^ mature dendritic cells in metastasis-containing SLNs [17]. On the other hand, according to Kohrt et al., DC and CD4^+^ T cell populations in axillary lymph nodes, but not in SLNs, predicted disease-free survival in breast cancer [7]. Accumulation of FOXP3^+^ regulatory T cells in SLNs was demonstrated to be associated with poor prognosis in breast and gastric cancer as well as in melanoma [13,14,18]. Furthermore, the presence of IDO^+^ cells in SLNs of melanoma patients correlated with unfavorable outcome [14,19].

Taken together, results on the immune competence of SLNs are controversial; while some studies suggest that these lymph nodes are immune deficient, “preconditioned” to host metastases by tolerogenic cells or suppressive factors deriving from the primary tumor [2,3,7,10], others seem to indicate that, on the contrary, SLNs are not suppressed, or may even be in an activated state due to stimulation by tumor antigens [4-6,9,11,12]. The presence of metastatic tumor in SLNs seems to be associated with elevated amount of regulatory T cells in several tumor types [8,13-15], which may indicate a state of immune suppression. However, results concerning other immune cell types are equivocal [9-13,15,20]. Few reports have addressed the prognostic potential of determining the immune status of SLNs [7,13,14,16-18] or associations of immune cell densities in SLNs with clinicopathologic factors [13,14,18].

Our previous studies investigating the prevalence of immune cell types in primary cutaneous melanomas identified infiltration by T lymphocytes expressing the activation markers CD25 and OX40, as well as the density of DC-LAMP^+^ mature DCs as prognostic factors [21,22]. These results suggest that the presence of activated T cells and antigen presenting DCs at the primary site could be a marker of a functional immune response against melanoma progression and influence the outcome of the disease. On the other hand, the density of FOXP3^+^ regulatory T lymphocytes in primary melanomas did not prove of prognostic significance [23]. In the present study we examined the prevalence of several immune cell types: OX40^+^ activated T lymphocytes, FOXP3^+^ regulatory T cells, DC-LAMP^+^ mature DCs and CD123^+^ plasmacytoid DCs in sentinel lymph nodes of melanoma patients. In selecting the markers to be studied we intended to include cell types the prevalence of which has proved of prognostic value in earlier studies on primary melanoma or sentinel nodes.

The main objectives of the study were i) to evaluate the immune status of SLNs in comparison to non-sentinel nodes, examining markers associated with immune activation (the presence of mature DCs and activated T cells) or those generally considered as signs of immune suppression (pDCs and Treg cells), and ii) to determine whether these immune parameters are predictive of the outcome of the disease in terms of tumor progression and patients’ survival.

## Methods

### Patients and samples

Archival tissue samples were obtained from 60 patients with cutaneous melanoma of >1 mm thickness who underwent sentinel lymph node dissection at the National Institute of Oncology, Budapest, between 1999 and 2001. The study was approved by the ethics committee of the institute and by the national Scientific and Research Ethics Council (ETT TUKEB). Patients did not receive any anticancer treatment prior to surgery. SLN dissection was performed at the time of the removal of the primary tumor in the majority of cases (n = 47), or 2 to 10 weeks (median: 4 weeks) later in patients whose primary melanoma had been operated in other institutions (n = 13). SLN biopsy was carried out using double labeling (^99m^Tc-albumin and patent blue dye) as previously described [24,25]. Histological evaluation of sentinel lymph nodes was performed on serial sections using hematoxylin-eosin staining and immunohistochemical labeling for MART-1/Melan-A.

In 38 cases there was no tumor progression detected in the follow-up period of 5 years; 28 of these patients presented with negative SLNs, 4 with isolated tumor cells or metastases <0.1 mm in diameter, 3 with metastases of 0.1–1.0 mm and 3 with metastases >1.0 mm according to the maximum diameter of the largest lesion. In 22 patients progression involving various organs was observed; 7 of them had tumor-free SLNs while 15 had positive nodes: 6 with metastases of 0.1–1.0 mm and 9 with metastases >1.0 mm in at least one of the SLNs examined. A total of 100 sentinel nodes (69 tumor-free and 31 tumor-positive) of these 60 patients were analyzed. Clinical and pathological characteristics are summarized in Table 2. Surviving patients had follow-up data for at least 5 years; none of the patients died of melanoma-unrelated causes within 5 years. Of the 22 patients showing disease progression, 17 had died by the end of the 5-year follow-up period while 5 patients were alive with (n = 3) or without (n = 2) disease.

**Table 2:** Patient and tumor characteristics

**Patient group**	**All patients**	**SLN-negative**	**SLN-positive**
Age – median (range)	54 (27–79)	54 (27–79)	54 (27–76)
Sex			
Male	27	16	11
Female	33	19	14
Tumor thickness (mm)			
1.01–2.0	29	21	8
2.01–4.0	20	8	12
>4.0	11	6	5
Tumor site			
Extremities	31	18	13
Trunk	29	17	12
Histologic type			
SSM	37	24	13
NM	19	10	9
ALM	4	1	3
Ulceration			
Absent	37	26	11
Present	20	9	11
Unknown	3	-	3
Progression in 5 years			
No	38	28	10
Yes	22	7	15
5-year survival (%)	43/60 (72)	31/35 (89)	12/25 (48)

SSM, superficial spreading melanoma; NM, nodular melanoma; ALM, acral lentiginous melanoma.

For a subset of the patients, lymph node samples from block dissection, performed because of positive SLN status 2 to 7 weeks (median: 3 weeks) following SLN biopsy, were also available (37 lymph nodes, two metastatic and 35 tumor-negative, from 7 patients).

### Immunohistochemical detection of immune cell types

Three*-*μm sections cut from formalin-fixed, paraffin-embedded samples were used. Immunohistochemistry was performed as described earlier [21-23], using monoclonal antibodies against FOXP3 (236A/E7; Abcam Inc., Cambridge, MA), OX40 (CD134) (BD Biosciences Eastern Europe, Heidelberg, Germany), CD123 (BD Biosciences), and DC-LAMP (CD208) (Beckman Coulter-Immunotech, Marseille, France), followed by polymer-conjugated secondary antibody (SS™ One-Step Polymer-HRP IHC Detection System, BioGenex, Fremont, CA), visualization with 3-amino-9-ethylcarbazole (Vector Laboratories, Inc., Burlingame, CA), and counterstaining with hematoxylin.

### Evaluation of the immune reactions

Slides were examined using a graticule of 10x10 squares, calibrated as 0.25 mm^2^ at 200× magnification. Counting was performed independently by two investigators, both blinded to the clinical information, and the mean value of their separate counts was used for the analysis. Sections were scanned at low magnification, and 5 areas with the highest density of positive cells (hot spots) were counted at 400x magnification. Staining for OX40 and CD123 could not be evaluated in one case each. In the case of patients with more than one SLN available, the mean labeled cell densities of all SLNs studied were also registered. For each marker, cutoff levels were set up based on the median of the given variable in the whole patient group, with minor adjustment for better discriminating power in the case of FOXP3, OX40 and CD123 (1900, 73, 480 and 936 cells/mm^2^ for FOXP3, OX40, CD123 and DC-LAMP, respectively), and the proportion of patients with a mean cell density higher than the cutoff level was determined.

### Statistical analysis

Comparisons between cell densities in different tumor groups was made using the Mann–Whitney U test and Kruskal-Wallis test, while χ^2^ test was used for comparing the proportions of samples with high density of labeled cells. Analysis of survival was performed by the Kaplan-Meier method, and the statistical analysis was carried out by the Mantel-Cox test. Univariate and multivariate Cox regression analyses were performed using mean immune cell densities, Breslow index and patients’ age as continuous variables, as well as location, histological type and ulceration of the tumors, SLN status and patient gender as categorical variables. All statistics were calculated using the BMDP Statistical Software Pack.

## Results

### Expression of the studied markers in sentinel lymph nodes

**Figure 1:**
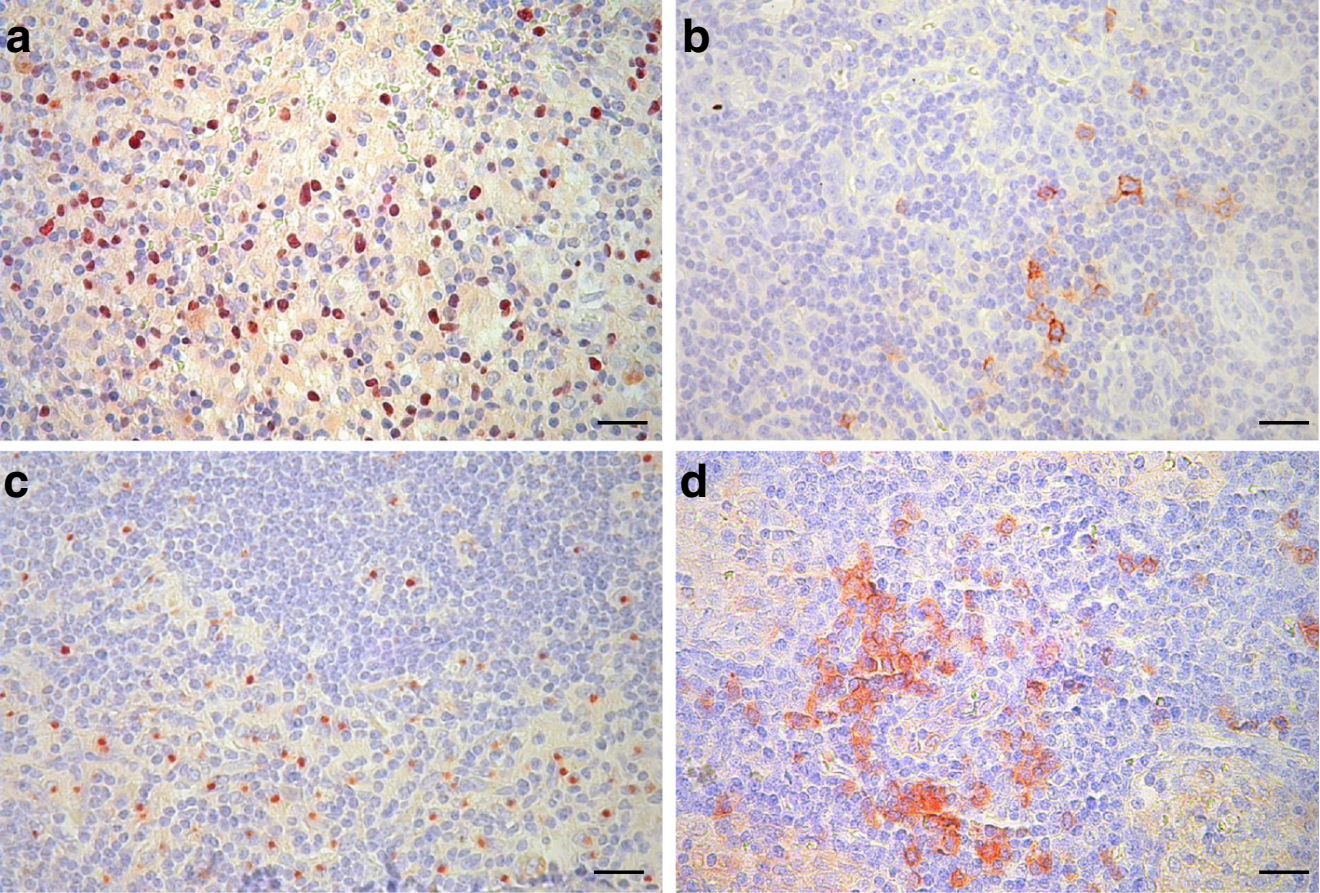
**FOXP3**^**+**^**(a), OX40**^**+**^**(b), DC-LAMP**^**+**^**(c) and CD123**^**+**^**cells (d) in melanoma sentinel lymph nodes (bars: 20 μm).**

Using immunohistochemical labeling with antibodies against marker antigens, we found varying amount of the four immune cell types studied in the lymph node samples. The majority of lymphocytes exhibiting nuclear staining for FOXP3, as well as those showing membrane staining for OX40, were distributed diffusely in the T-cell areas of the paracortex (Figure 1a,b). Mature dendritic cells, showing the characteristic juxtanuclear dot-like staining for the DC-LAMP marker, were also located predominantly in the paracortical regions of lymph nodes, surrounding T-cell areas (Figure 1c). CD123^+^ plasmacytoid DCs appeared either scattered or as aggregates in the paracortex, mostly around high endothelial venules, also showing positive staining for CD123 (Figure 1d). In some of the metastatic nodes, especially in cases with large tumor deposits, localization of OX40^+^ lymphocytes as well as DC-LAMP^+^ dendritic cells in the vicinity of metastases could be seen, occasionally infiltrating the tumors. This was less frequently observed in the case of FOXP3^+^ lymphocytes or CD123^+^ pDCs. Quantitative analysis of immunohistochemistry results, evaluating 5 areas with the highest density of positive cells at high magnification in each sample, revealed that FOXP3^+^ lymphocytes were the most abundant (mean ± SD, 2003 ± 720 cells/mm^2^), followed by DC-LAMP^+^ mature dendritic cells (938 ± 280 cells/mm^2^) and CD123^+^ pDCs (650 ± 434 cells/mm^2^), while OX40^+^ activated T lymphocytes were the least numerous (100 ± 68 cells/mm^2^). No correlation was found between density values of the different cell types.

### Comparison of immune cell densities in sentinel vs. non-sentinel nodes and in metastatic vs. tumor-free SLNs

**Figure 2:**
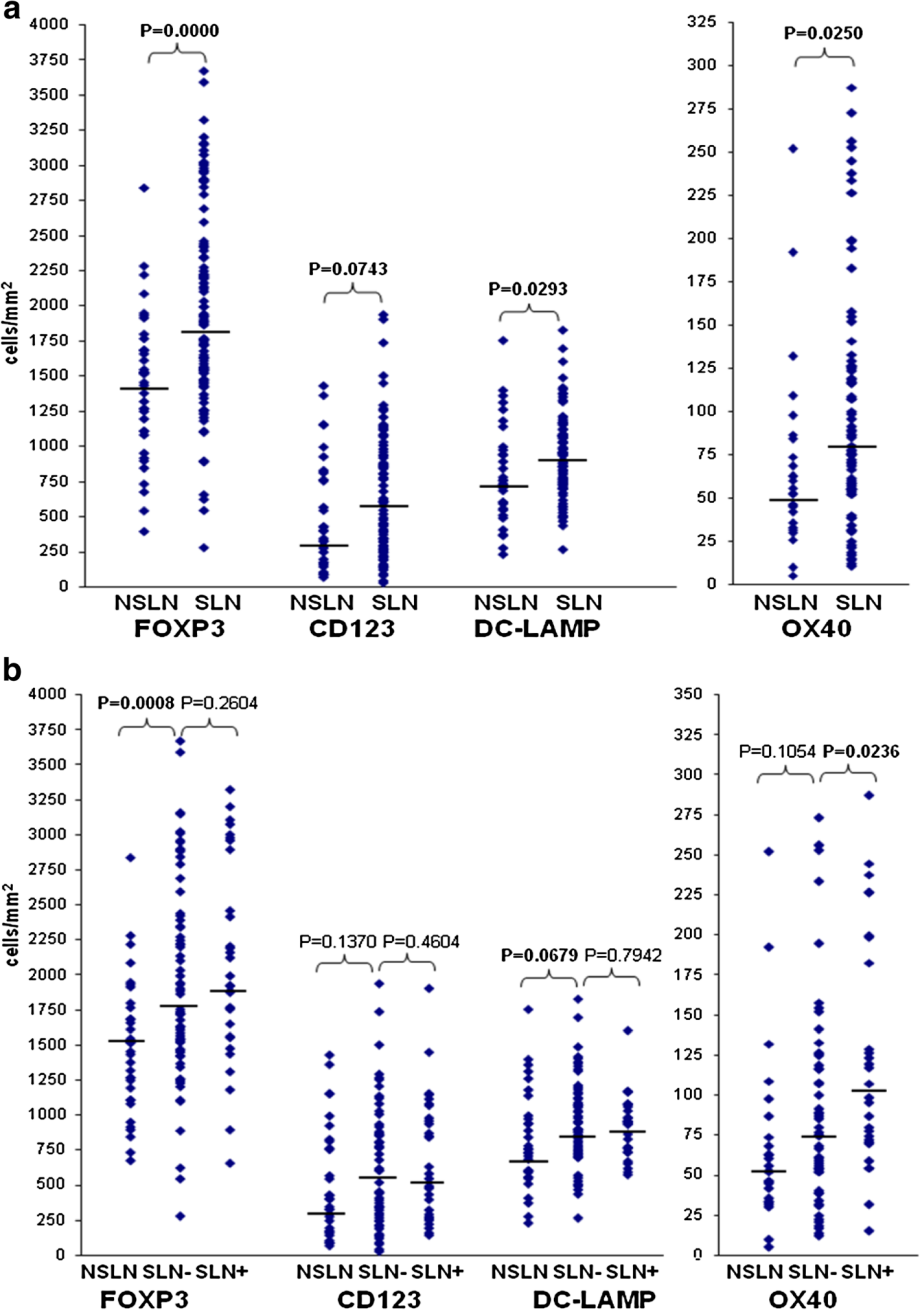
**Density of immune cell types a) in non-sentinel (n = 37) and sentinel (n = 100) lymph nodes; b) in tumor-negative NSLNs (n = 35), and tumor-negative (n = 69) or -positive (n = 31) SLNs.** Dots represent individual values; horizontal bars are medians.

For a subset of the above patients, lymph node samples from block dissection, performed because of positive SLN status, were also available (37 lymph nodes from 7 patients). Compared to SLNs, these nodes showed lower density of cells labeled for FOXP3, OX40 and DC-LAMP, while a difference of borderline significance was noted in the number of CD123^+^ plasmacytoid DCs (Figure 2a). The same trends, although with less significant differences, were observed when only tumor-free lymph nodes of the SLN (n = 69) and NSLN group (n = 35) were considered (Figure 2b). In these studies sentinel nodes from all patients (35 SLN-negative and 25 SLN-positive) vs. non-sentinel nodes from 7 patients (all SLN-positive) were evaluated. Similar results were obtained when sentinel nodes from only the SLN-positive patients were used in the comparisons (data not shown). Moreover, individual comparison of mean densities of labeled cells in SLNs and NSLNs of the 7 patients with available NSLN samples (Table 3) shows that in the majority of patients SLN values are higher or comparable to NSLN values for all immune cell types.

**Table 3:** **Comparison of mean immune cell densities in SLNs and NSLNs for 7 patients with available SLN and NSLN samples (mean density values for lymph nodes from each patient; cells/mm**^**2**^**)**

**Patient no.**	**FOXP3**		**OX40**		**CD123**		**DC-LAMP**	
	**SLN**	**NSLN**	**SLN**	**NSLN**	**SLN**	**NSLN**	**SLN**	**NSLN**
1	1958	957	193	65	232	280	818	660
2	1925	1477	119	192	1450	621	957	648
3	2205	1060	15	60	222	214	573	1038
4	1562	1450	117	29	938	242	592	657
5	2123	2086	245	192	1154	566	1059	384
6	2814	1747	61	33	244	225	925	744
7	1768	1633	54	60	395	1040	1171	989

Comparison of sentinel lymph nodes diagnosed as tumor-positive (n = 31) or -negative (n = 69) according to histological and immunohistochemical analysis showed higher mean density of OX40^+^ lymphocytes in positive nodes compared to negative ones, while no significant difference was observed in the amount of the other cell types studied (Figure 2b).

### Associations of immune cell densities in sentinel nodes with clinicopathologic factors

Evaluating the mean labeled cell densities in SLNs, no associations with patient or tumor parameters (Breslow index, histological type, ulceration and location of the tumors, patients’ age and gender) were found for any of the immune cell markers studied. Analysis of the proportion of patients with mean cell densities higher than the cutoff level, with respect to the above parameters, revealed no association for any of these parameters either, except an elevated ratio of cases with high mean CD123^+^ cell density in younger patients (Additional file 1).

### Analysis of disease progression and survival according to immune cell densities in the sentinel nodes

**Figure 3:**
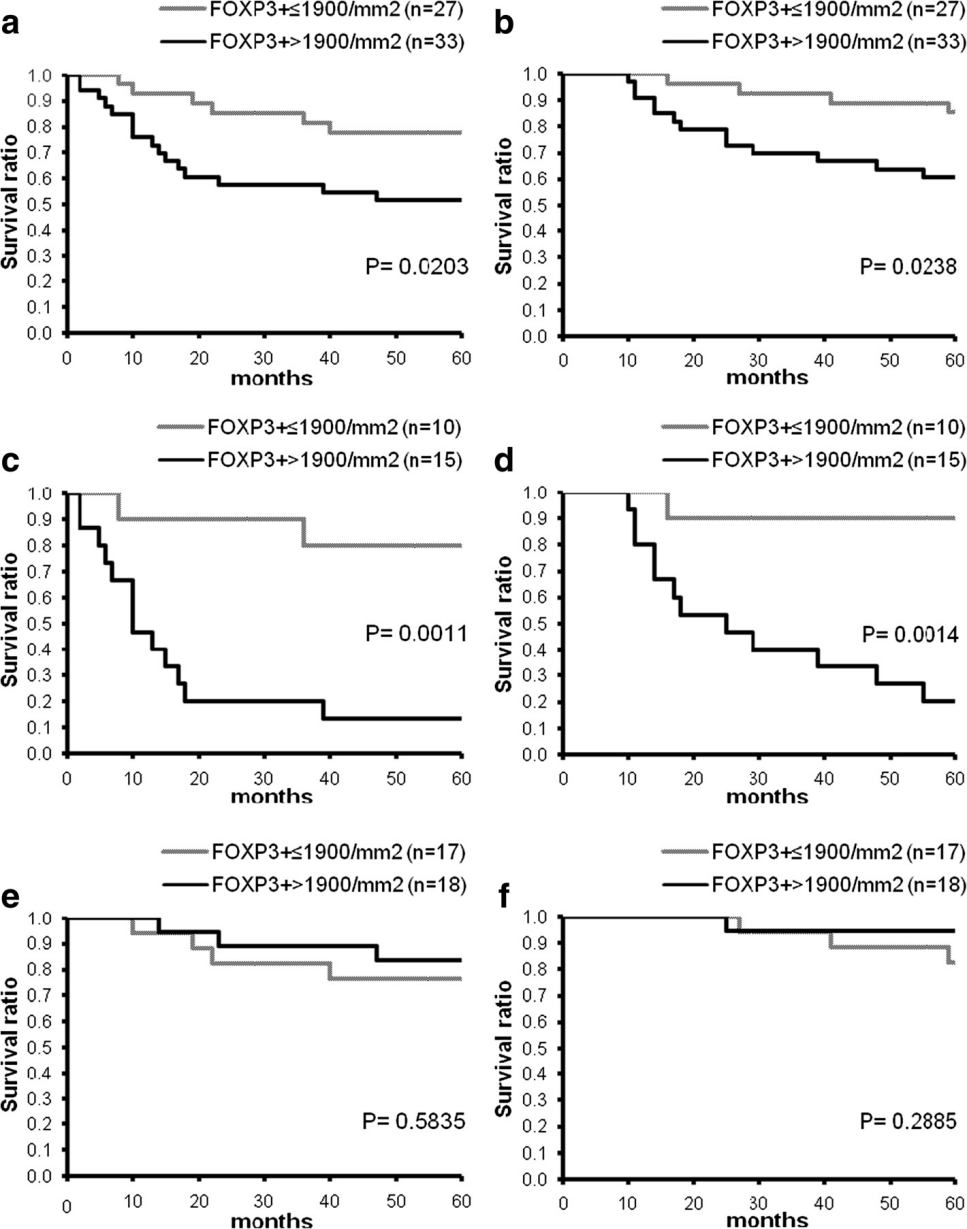
**Kaplan-Meier curves of progression-free (a,c,e) and overall survival (b,d,f) for melanoma patients (a,b: all patients; c,d: positive SLN status; e,f: negative SLN status) subdivided according to mean SLN FOXP3**^**+**^**cell density.**

Kaplan-Meier analysis of survival according to the mean density of FOXP3^+^ lymphocytes in SLNs revealed that high number of these cells was associated with significantly shorter progression-free and overall survival (Figure 3a,b). Analyzing cases separately for patients with positive and negative SLN status revealed that the above findings were entirely due to the prominent differences observed in the subgroup of sentinel-positive patients (Figure 3c,d), while no such variance could be seen in the sentinel-negative patient group (Figure 3e,f). The amount of the other immune cell types studied did not show significant correlation with survival.

The potential prognostic effect of immune cell densities evaluated as continuous variables, together with other clinicopathologic factors (thickness, location, histological type and ulceration of the primary tumor, SLN status, patient age and gender), was also evaluated using Cox’s proportional hazards model. In univariate analysis of all cases, ulceration, Breslow index, SLN status and histological type were found significantly associated with both progression-free and overall survival (P = 0.000, P = 0.001, P = 0.003 and P = 0.033 for PFS; P = 0.000, P = 0.000, P = 0.002 and P = 0.028 for OS, respectively). Multivariate analysis identified ulceration, Breslow index, SLN status and patient gender as significant independent predictors of survival (P = 0.000, P = 0.005, P = 0.005 and P = 0.017 for PFS; P = 0.000, P = 0.005, P = 0.016 and P = 0.008 for OS, respectively). The amount of the studied cell types in SLNs did not prove predictive of either progression-free or overall survival. Analyzing only patients with positive SLN status, however, yielded significant or near significant associations between mean FOXP3^+^ cell density and survival (univariate analysis: P = 0.054 and P = 0.038; multivariate analysis: P = 0.046 and 0.069 for PFS and OS, respectively), with ulceration, Breslow index and patient gender as other significant covariates. In the case of SLN-negative patients, only histological type and ulceration of the tumor showed association with progression-free and overall survival.

## Discussion

In this retrospective study we determined the density of several immune cell types: OX40^+^ activated T lymphocytes, FOXP3^+^ regulatory T cells, DC-LAMP^+^ mature dendritic cells and CD123^+^ plasmacytoid DCs in sentinel lymph nodes of patients with malignant melanoma. Density values of each cell type in SLNs vs. lymph nodes obtained from block dissection, as well as those in tumor-free vs. metastatic SLNs were compared. Furthermore, results were evaluated with regard to associations with patient and tumor parameters and the outcome of the disease.

In our cohort of 100 sentinel nodes, FOXP3^+^ lymphocytes proved the most abundant cell type, greatly outnumbering OX40^+^ activated T cells. This finding could be considered a sign of immunosuppression. However, OX40 is a very specialized marker expressed primarily by recently activated CD4^+^ T lymphocytes showing preferential accumulation at tumor sites in cancer patients and is thought to represent tumor antigen specific T cells [26,27]. Since it is not a general marker of all activated T cells, the expression of OX40 is expected to be restricted. Moreover, most immune cell types studied were present in higher amount in the sentinel nodes compared to 37 non-sentinel nodes from a subset of the patients, not only FOXP3^+^ Tregs but also OX40^+^ activated T cells and DC-LAMP^+^ mature DCs.

Our results were mainly based on interindividual comparisons of labeled cells in SLNs vs. NSLNs. We did not perform statistical analysis comparing SLN-NSLN pairs of the same patients because of the low number of cases with available NSLN samples. Moreover, since it was a retrospective study, we did not have access to non-sentinel nodes of sentinel-negative patients. On the other hand, in our study, similarly to others comparing sentinel and non-sentinel nodes from the same patients, NSLNs derived from the same lymph node basin as the SLN. Therefore, they were likely also affected by the primary tumor albeit to a lesser degree than the SLN. Nevertheless, our results indicate a state of functional activity in the sentinel lymph nodes, or at least no unambiguous signs of immune suppression compared to NSLNs.

The question whether sentinel nodes are immunologically competent or suppressed is a matter of debate, with no consensus reached yet. According to Cochran’s group, SLNs draining melanoma and breast cancer show evidence of immunosuppression, with lower number of paracortical dendritic cells (DCs) and lower expression of costimulatory molecules compared to NSLNs [2,3]. Other studies examining various DC markers, however, found either higher amount of DCs in SLNs or no difference from NSLNs or control lymph nodes in melanoma, breast, gastric or oral cancers [4-6,9-12] (Table 1). One reason for this discrepancy could be the relatively small sample size in many of the studies comparing SLNs and NSLNs, hampering the drawing of reliable conclusions. Also, while some of these investigations evaluated metastatic and tumor-free SLNs separately, others did not make such distinction which makes the results difficult to compare. Nevertheless, most comparative studies mentioned above, including the few involving higher case numbers, seem to indicate that sentinel lymph nodes are not suppressed (Table 1). It should be noted, however, that signs of immune activation and those of dysfunction can be detected at the same time in some of the studies, including ours. Moreover, the balance of activation and suppression most probably changes with time, parallel to tumor progression.

Analysis of our results according to tumor positivity of sentinel nodes resulted in no significant difference in the case of three of the four markers studied, including FOXP3, in contrast to some [14,15], but not all [10] other studies on melanoma SLNs (Table 1). The reason for this discrepancy is not known, but may partly be explained by methodological differences. On the other hand, in our present study positive nodes contained higher amount of OX40^+^ activated T lymphocytes compared to negative ones, which may be related to in situ activation of T cells recognizing tumor antigens. In accordance with this finding, a previous report on axillary lymph nodes of breast cancer patients revealed preferential accumulation of OX40^+^ cells only in positive nodes [27].

We did not find significant difference between metastatic and tumor-free SLNs in the density of DC-LAMP^+^ mature dendritic cells or CD123^+^ plasmacytoid DCs. Results concerning dendritic cell subtypes in SLNs are contradictory in the case of melanoma and other tumor types as well (Table 1). A recent paper described decreased number of CD11c^+^ dendritic cells but elevated amount of CD86^+^ mature DCs in positive compared to negative sentinel lymph nodes in melanoma [15]. In breast cancer, mature DCs detected by the CD83 or DC-LAMP marker were present in lower amount in tumor-positive SLNs compared to tumor-negative ones while the density of DCs stained for CD1a was similar [9,12]. On the other hand, there was no difference in the number of either S100^+^ or DC-LAMP^+^ DCs in metastatic vs. tumor-free SLNs in gastric carcinoma [11,13]. The prevalence of plasmacytoid DCs (pDCs) was found more prominent in tumor-containing SLNs in one study on melanoma [20] while no difference was detected between metastasis-positive and -negative SLNs in another [10] or in breast cancer [9].

In our patient cohort, density of DC-LAMP^+^ mature DCs, CD123^+^ plasmacytoid DCs and OX40^+^ activated T cells in sentinel nodes did not show significant association with disease progression or survival. No previous studies have been reported on a potential prognostic effect of the amount of pDCs or activated T cells in sentinel nodes in any tumor types. In a study on tumor-positive SLNs in melanoma, the accumulation of DC-LAMP^+^ DCs was shown to have a statistically significant but weak impact on survival, and did not prove independent prognostic factor [17]. On the other hand, infiltration of primary melanomas by DC-LAMP^+^ mature dendritic cells and OX40^+^ activated T cells proved strong independent predictors of survival according to our previous studies [21,22]. The above findings on the more prominent prognostic effect of these cell types at the site of the primary melanoma may indicate the importance of immune cells residing in the primary tumor in the development of antitumor immune response.

On the other hand, the amount of FOXP3^+^ cells infiltrating primary melanomas did not show association with the outcome of the disease according to our previous study [23]. In contrast to these results, our present findings indicate that quantification of FOXP3^+^ cells in SLNs may provide information with prognostic impact. High mean density of these cells was associated with significantly shorter progression-free and overall survival. A novel, intriguing finding of our study is that high density of FOXP3^+^ cells in sentinel nodes was associated with disease progression and shorter survival only in cases with positive SLN status. Moreover, univariate and multivariate Cox regression analyses evaluating mean cell density values as continuous variables showed associations with survival only in the case of positive SLN status and not in patients with negative SLNs or in the whole patient group. A recent study by Speeckaert et al., not evaluating cases with different SLN status separately, also could not prove the independent prognostic effect of FOXP3^+^ cells in sentinel nodes of melanoma patients [14]. Although the sentinel-negative but progressing cases in our study represented a minority of the samples (7 patients), which in theory might have influenced the result of such comparisons, differences in PFS and OS were much larger in the SLN-positive group compared to those in the whole cohort. These results indicate that Tregs may exert their suppressor function more efficiently in metastatic nodes, at least at a degree that would be reflected in influencing the outcome of the disease. Reasons for this difference could include the relative abundance of Tregs in positive nodes as suggested by some studies [8,13-15], or an elevated activity of tumor-specific immune reactions in the presence of tumor antigens.

Priming of antitumor T-cell responses is generally believed to take place primarily in draining lymph nodes via cross presentation of tumor antigens by dendritic cells capturing antigens at the primary tumor site and migrating to lymph nodes. However, in experimental models direct priming of T lymphocytes by tumor cells in the lymph nodes has been described [28,29]. In melanoma patients, precursor frequency of peripheral blood CD8^+^ T cells recognizing melanocyte differentiation antigen epitopes was found increased in stage III and IV vs. stage I and II, indicating the importance of the development of lymph node metastases in triggering T-cell mediated antitumor immunity [30]. On the other hand, it has also been shown that T-cell activation by dendritic cells may occur extranodally, at tumor sites [31]. In accordance with this finding, a study analyzing T-cell clonotypes revealed that the majority of T cells in tumor-negative SLNs are not clonally expanded [32], in contrast to primary melanomas and metastatic lesions showing the presence of multiple clonotypic TCR transcripts [32,33]. In the light of these data, the results of our present and previous studies could be interpreted as suggesting the prognostic importance of immune markers in tumoral compartments of melanoma patients, both in the primary tumor and in metastatic sentinel lymph nodes.

## Conclusion

We have shown that the prevalence of the studied immune cells in sentinel lymph nodes is higher compared to non-sentinel ones, which is compatible with the hypothesis of functional competence of SLNs, supported by the majority of previous studies. Some of the changes seem to occur before the arrival of tumor cells, while the amount of OX40^+^ activated T cells was found elevated in metastatic SLNs. The density of FOXP3^+^ lymphocytes showed association with disease progression and shorter survival in sentinel-positive patients, while the other immune cell types studied did not prove of prognostic significance. These results, together with our previous findings on the prognostic value of activated T cells and mature DCs infiltrating primary melanomas, suggest that immune activation-associated markers in the primary tumor may have a higher impact than those in SLNs on the prognosis of the patients. On the other hand, FOXP3^+^ cell density in metastatic SLNs, but not in tumor-negative ones or in primary melanomas, was found predictive of the outcome of the disease.

Our results were based on a retrospective analysis of lymph node samples of a sizeable group of melanoma patients, however, evaluating sentinel-positive and negative cases separately according to disease progression or survival yielded more limited case numbers. To obtain a more complete view on the immune characteristics of these lymph nodes and on the prognostic value of these immune markers, prospective studies on larger patient cohorts are warranted.

## Competing interests

The authors declare that they have no competing interests.

## Authors’ contributions

AM and TS participated in immunohistochemistry studies, evaluation of results and helped in drafting the manuscript. GL provided data on patient follow-up. VP and SH carried out pathological evaluation of samples. IG performed statistical analysis of data. AL conceived the study, participated in its design and execution, and drafted the manuscript. All authors read and approved the final manuscript.

## Supplementary Material

Additional file 1 Proportion of patients with significant mean SLN immune cell density in groups with different patient and tumor characteristics. Click here for file
