# Supplementary material for: Immune cell profile of sentinel lymph nodes in patients with malignant melanoma – FOXP3+ cell density in cases with positive sentinel node status is associated with unfavorable clinical outcome
Source: J Transl Med. 2013 Feb 18;11:43. doi: 10.1186/1479-5876-11-43 (PMC3598232; doi:10.1186/1479-5876-11-43)
Supplement: Additional file 1 — Proportion of patients with significant mean SLN immune cell density in groups with different patient and tumor characteristics. [file 1479-5876-11-43-S1.pdf]

**Additional file 1. Proportion of patients with significant mean SLN immune cell density in groups with different patient and tumor characteristics**

| | Patient no. | FOXP3 (>1900 cells/mm$^2$) | | OX40 (>73 cells/mm$^2$) | | CD123 (>480 cells/mm$^2$) | | DC-LAMP (>936 cells/mm$^2$) | |
|---|---|---|---|---|---|---|---|---|---|
| | | No. (%) | P | No. (%) | P | No. (%) | P | No. (%) | P |
| **All patients** | **60** | 33 (55) | | 34 (58) | | 34 (58) | | 30 (50) | |
| **Age** | | | | | | | | | |
| **<53 years** | **30** | 15 (50) | | 18 (60) | | 21 (72) | | 13 (43) | |
| **>53 years** | **30** | 18 (60) | n.s. | 16 (55) | n.s. | 13 (43) | **0.0238** | 17 (57) | n.s. |
| **Sex** | | | | | | | | | |
| **Male** | **27** | 17 (63) | | 18 (69) | | 14 (52) | | 15 (56) | |
| **Female** | **33** | 16 (48) | n.s. | 16 (48) | n.s. | 20 (62) | n.s. | 15 (45) | n.s. |
| **Location** | | | | | | | | | |
| **Extremities** | **31** | 18 (58) | | 20 (65) | | 16 (52) | | 13 (42) | |
| **Trunk** | **29** | 15 (52) | n.s. | 14 (50) | n.s. | 18 (64) | n.s. | 17 (59) | n.s. |
| **Type**[1] | | | | | | | | | |
| **SSM** | **37** | 19 (51) | | 22 (61) | | 21 (58) | | 19 (51) | |
| **NM** | **19** | 10 (53) | n.s. | 9 (47) | n.s. | 13 (68) | n.s. | 11 (58) | n.s. |
| **Thickness (mm)** | | | | | | | | | |
| **1.01-2.0** | **29** | 14 (48) | | 13 (46) | | 16 (57) | | 15 (52) | |
| **2.01-4.0** | **20** | 11 (55) | | 14 (70) | | 13 (65) | | 8 (40) | |
| **>4.0** | **11** | 8 (73) | n.s. | 7 (64) | n.s. | 5 (45) | n.s. | 7 (64) | n.s. |
| **Ulceration**[1] | | | | | | | | | |
| **Absent** | **37** | 19 (51) | | 18 (50) | | 21 (58) | | 20 (54) | |
| **Present** | **20** | 12 (60) | n.s. | 13 (65) | n.s. | 12 (60) | n.s. | 10 (50) | n.s. |
| **SLN status** | | | | | | | | | |
| **Negative** | **35** | 18 (51) | | 19 (54) | | 19 (56) | | 16 (46) | |
| **Positive** | **25** | 15 (60) | n.s. | 15 (62) | n.s. | 15 (60) | n.s. | 14 (56) | n.s. |

[1]ALM (n=4) cases, and 3 cases with ulceration unknown are not shown; n.s., not significant;
SSM, superficial spreading melanoma; NM, nodular melanoma; ALM, acral lentiginous melanoma
